# Employing Relative Entropy Techniques for Assessing Modifications in Animal Behavior

**DOI:** 10.1371/journal.pone.0028241

**Published:** 2011-12-02

**Authors:** Minoru Kadota, Eric J. White, Shinsuke Torisawa, Kazuyoshi Komeyama, Tsutomu Takagi

**Affiliations:** 1 Department of Fisheries, Faculty of Agriculture, Kinki University, Naka-machi, Japan; 2 Department of Physics, University of Cincinnati, Cincinnati, Ohio, United States of America; 3 Faculty of Fisheries, Kagoshima University, Shimoarata Kagoshima, Japan; Universita' del Piemonte Orientale, Italy

## Abstract

In order to make quantitative statements regarding behavior patterns in animals, it is important to establish whether new observations are statistically consistent with the animal's equilibrium behavior. For example, traumatic stress from the presence of a telemetry transmitter may modify the baseline behavior of an animal, which in turn can lead to a bias in results. From the perspective of information theory such a bias can be interpreted as the amount of information gained from a new measurement, relative to an existing equilibrium distribution. One important concept in information theory is the relative entropy, from which we develop a framework for quantifying time-dependent differences between new observations and equilibrium. We demonstrate the utility of the relative entropy by analyzing observed speed distributions of Pacific bluefin tuna, recorded within a 48-hour time span after capture and release. When the observed and equilibrium distributions are Gaussian, we show that the tuna's behavior is modified by traumatic stress, and that the resulting modification is dominated by the difference in central tendencies of the two distributions. Within a 95% confidence level, we find that the tuna's behavior is significantly altered for approximately 5 hours after release. Our analysis reveals a periodic fluctuation in speed corresponding to the moment just before sunrise on each day, a phenomenon related to the tuna's daily diving pattern that occurs in response to changes in ambient light.

## Introduction

Understanding the movement patterns of animals is crucial for their proper management and conservation. In particular, the analysis of telemetry data provides valuable insight into the movement, stock structure, and environmental preferences of individually tagged animals. In order to properly understand the relationship between an animal's behavior and its environment, it is essential that researchers determine the possible effects that transmitter attachment and presence can have on equilibrium behavior and physiology. For instance, the attachment of a transmitter can induce stress in the animal, thereby interrupting its normal foraging behavior. In such cases the speed of an animal may be a good indicator of non-equilibrium behavior. Measurements of speed distributions for newly tagged animals can be significantly different from speed distributions under normal behavior. However, mild stress may not always be reflected by statistically significant changes in the speed, even though an observer confidently asserts that the animal is not behaving normally.

The key for a successful study of an animal's behavior is to ensure that any data used for analysis is indicative of its natural behavior. Distress from capture, along with the physiological impacts due to the attachment and presence of a transmitter, can result in stress that modifies an animal's baseline behavior [1], [2], [3]. James *et al*. [4] suspected some temporally short-term tagging effects on leatherback turtles at sea, and thus excluded from their data all results from the first week of tagging. In order to ensure that certain species of fish have properly recovered from the effects of anesthetics, attachment procedure, and transmitter presence, some studies have suggested that researchers would be well advised to exercise caution when analyzing data collected within the first twenty-four hours of transmitter attachment [5]. However, when it comes to quantifying the effects of external stress on animal behavior, no sophisticated or sufficiently quantitative methods have yet been established. Thus we turn to the question: how can one quantitatively distinguish the difference between stress-induced, non-equilibrium distributions and those of normal behavior ?

Often it is the case that an equilibrium (reference) distribution is constructed from past records of observation. Upon making a new measurement, one is generally interested in the amount of information gained from the measured (observed) distribution. However, when the newly observed distribution does not differ significantly from the reference distribution, no meaningful information is gained. In this case, the inaccessibility of new information serves not as a statement about any inherent utility of the newly measured distribution, but rather that the observed distribution does not significantly differ from the reference distribution.

There are currently several test statistics which are used to quantify the similarities between two distributions, including the *t*- and *F*-tests for Gaussian distributions, and the Kolmogorov-Smirnov test for generalized distributions [6]. Bayesian methods often employ null hypothesis testing, an approach that has been criticized for its inherent subjectivity and emphasis on decision-making statistics [7].

The purpose of this paper is to introduce the readers to the use of relative entropy techniques as a method of quantifying time-dependent differences between observed data and equilibrium. Relative entropy techniques are robust, compelling, and can be applied to many physical situations. Since relative entropy is sensitive to the higher-order moments of a distribution, and not just changes in the mean and variance, it has the major advantage of more completely capturing probabilistic information [8]. For example, relative entropy techniques can be used to detect a divergence between an observed distribution and equilibrium that may not affect low-order moments, such as a time-skewness introduced by difficulties in detection. Although the relative entropy is not a true metric in the mathematical sense, another useful property is that it can be intuited as an effective distance between two probability distributions, in the sense that 1) it is always positive, 2) it is zero if and only if the two distributions are identical, and 3) it increases as the distributions diverge [9], [10].

Despite the concern raised by the increased use of telemetry techniques, very little is known about the effects of tagging devices, and even less is known about the effects on fishes [11], [12], [13], [14], [15]. In this paper we introduce relative entropy techniques as a method for assessing factors that can influence and modify animal behavior. In the Methods section we define relative entropy, give a brief overview of its properties as applied to generalized probability distributions, then specialize the definition for the case of Gaussian distributions. In the Results section we analyze the effects that transmitter attachment and presence can have on the behavior of Pacific bluefin tuna. In the final section we conclude with a discussion of the results.

## Methods

### Relative Entropy

When performing a statistical data analysis, one often wishes to know by how much two probability distributions differ from each other. In information theory, the most common measure for doing this is the relative entropy. Consider a random variable *x* with a probability distribution function of 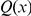. Following some measurement, we revise our estimate from 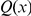 to 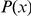. The change in the probability function represents a measure of the amount of information introduced as a result of the measurement. The relative entropy 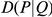 quantifies the change in information as an effective distance between the two probability distributions, given by
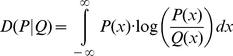
(1)


There exist many excellent reviews of the relative entropy in literature [10], [16], [17], [18]. Although this parameter, also known as the Kullback-Leibler divergence, is not a true metric, it serves as an effective distance between the distributions *P(x)* and *Q(x)*. The relative entropy is always non-negative, and vanishes if and only if *P = Q* for all *x*. Although the relative entropy is not symmetric under the exchange of *P* and *Q*, nor does it satisfy the triangle inequality, these relations are satisfied to a good approximation for 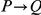.

A useful criterion in the analysis of empirical data is that the results not be dependent on the coordinate system used to describe a particular behavioral pattern, which often depends upon a choice of metric. A powerful feature of the relative entropy is that it is invariant under a change of coordinate systems. Consider a smooth, invertible transformation from *x* to *y* described by the function 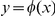. Since the relation 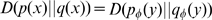 is always satisfied for such a re-parameterization, the relative entropy remains invariant under coordinate transformations [8], [19]. Thus, we are guaranteed that the difference between two probability distributions is always described by a single measure, regardless of the coordinate system. We further examine the significance of coordinate invariance as it applies specifically to the case of telemetry data in the Discussion section.

### Relative Entropy for Gaussian Distributions

An analytical expression may be obtained for the relative entropy in the case that 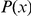 and 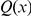 are Gaussian. Let us assume that the first and second moments of these distributions are denoted by 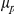, 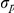, 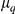, and 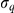, respectively. Given the standard form of a Gaussian distribution [20], it is straightforward to show that the relative entropy takes the form
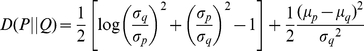
(2)Notice that the relative entropy can be decomposed into a set of uncorrelated components. The term in square brackets reflects any difference in the variances between the two distributions, and is often referred to as the dispersion component [8], [19]. When the variance of the observed distribution is small compared to that of the reference distribution, the relative entropy is dominated by this first term. In this case, the dispersion represents the reduction in uncertainty of the random variables as a result of the observation process.

Alternatively, when the means of the two distributions are large relative to the variance of the reference distribution, the relative entropy is dominated by the last term, often referred to as the signal component. The significance of the signal term can be better understood with the help of concrete example. Suppose that the mean speed for an oceanic bluefin tuna is 0.8 m/s, with a variation of 0.1 m/s. A new observation yields a measured speed of 1.2 m/s, with a variation of 0.1 m/s. Clearly, the utility of this new observation derives not from any improvement in the variation, since they are both equal to 0.1 m/s, but rather from a shift in the mean value. Assuming that both distributions are Gaussian, we can use Equation (2) to compute the relative entropy. Since only the signal term contributes to the relative entropy in this case, we can plug the given speeds and variance directly into the second term to get a value of 8 nats (logarithmic units). Thus, the distance between the two distributions is composed entirely by the difference in their central tendencies.

### Ethics Statement

This study (No. 2010-26) is conducted with approval by the Faculty of Agriculture, Kinki University, located in Higashi-Osaka, Japan. All experiments were conducted in accordance with Japanese Governmental law (No. 105), as well as the guidelines published by the Science Council of Japan concerning the appropriate treatment of animals in life science research.

### Procedure

We use data collected from an experiment conducted in the waters offshore of Kochi Prefecture, Japan. Three Pacific bluefin tuna were captured within a submerged net-cage with a diameter of 50 meters, then subsequently tagged with a data-logger package consisting of a data-logger and recovery system. The data-logger package is surgically attached to the right side of the body below the anterior lobe of the dorsal fin, using two plastic attachment wires connected to a time-release mechanism. The tags are affixed via an attachment plate aligned along the lateral line of the tuna's body. After two hypodermic needles are pushed through the dorsal musculature, a plastic wire is used to secure the attachment plate in place. Data collection begins when the tuna are returned to the submerged net-cage. Due to limited memory capacity and battery life, the tuna's speed was recorded at uniform 1-second intervals for a continuous span of forty-eight hours.

In order to ensure that any telemetry data collected in this experiment is indicative of the tuna's natural behavior, it is essential to quantify any physiological impacts incurred by the capture and attachment of the data-logger package. Since the speed of a bluefin tuna serves as a good criterion for discriminating non-equilibrium behavior, we apply relative entropy techniques to quantify the effects of stress by measuring the difference between the speed distributions of newly tagged tuna and equilibrium. The procedure is as follows: first, all time series corresponding to step-lengths measured at 1-second intervals are collected into bins of 10-minute intervals. For each bin 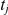 we compute the probability density function 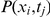, where 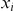 is the step-length and 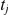 is the time corresponding to the *j*-th interval. The reference distribution 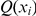 is then formed by averaging 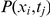 over all time intervals. Assuming the data is normally distributed, we perform a chi-squared goodness-of-fit test using the null hypothesis. Since the null hypothesis cannot be rejected to a significance level of 5%, we suppose that 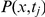 and 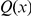 are Gaussian distributions of dimension one.

In order to definitively state that a tuna's behavior is no longer affected by trauma, we must establish at which point in time an observed data set is statistically indistinguishable from the reference distribution. A *t*-test is commonly employed to determine if the mean values of the observed and reference distributions are statistically consistent. In Appendix S1, we discuss the connection between the *t*-test and the signal component of the relative entropy for Gaussian distributions. That such a relation exists should come as little surprise: any difference between the mean values of an observed and reference distribution will contribute to the overall “distance” between them, which in turn establishes the amount of information provided by the observation. For the concrete example provided above, it is clear that the signal component of the relative entropy vanishes when the two means are equal.

## Results

**Figure 1:**
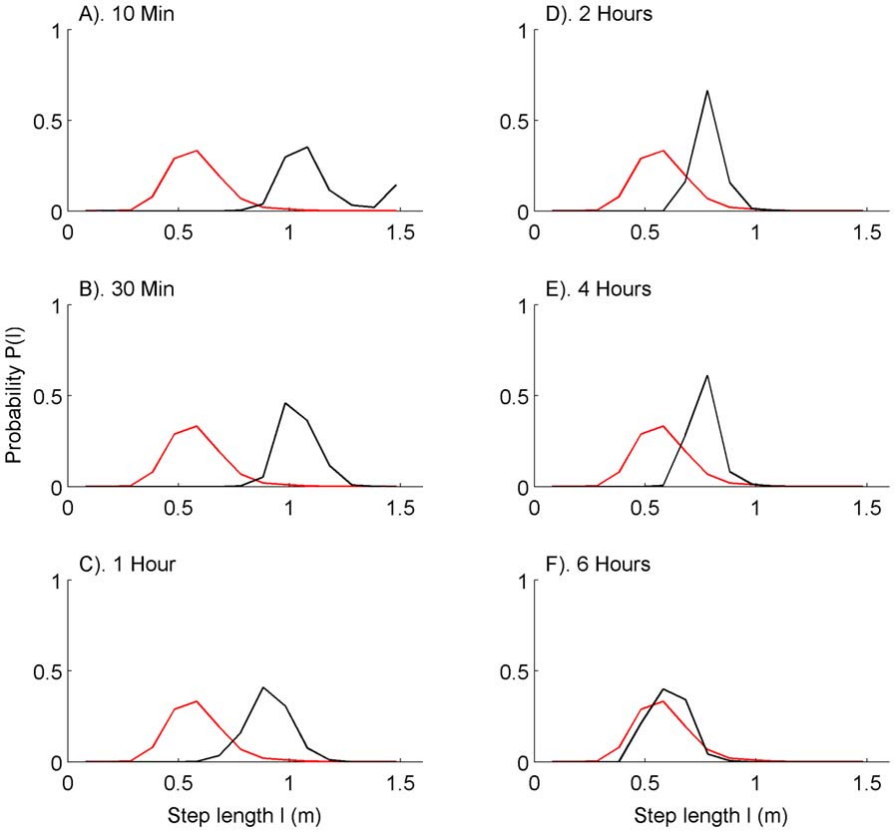
Behavior of observed step-length distributions over time. Observed (black) and reference (red) probability density functions of the step length 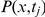 and 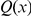 at various times. Observed distributions 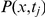 were calculated at intervals of 10 minutes, 30 minutes, 1 hour, 4 hours, and 6 hours after release. This figure demonstrates how the observed distributions 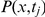 approaches the reference distribution 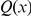 over time.

**Figure 2:**
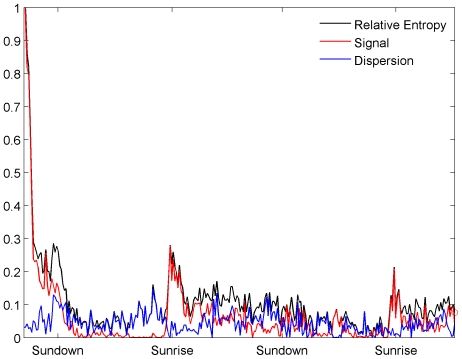
Relative entropy distribution after release. Relative entropy 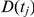 (black), signal component (red), and dispersion component (blue), plotted versus hours after release. The periodic increases in the signal component correspond to the moment just before sunrise on each day.

We begin by comparing a newly observed speed distribution 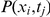 for a single bluefin tuna with that of a reference distribution 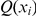, obtained by averaging 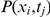 over all time intervals. In both cases, the distributions correspond to step-lengths measured at 1-second intervals and collected into bins of 10-minute intervals, as described above in the Methods section. In Figure 1 we show the observed probability distribution function for 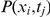 calculated in each bin 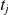, along with the re-averaged reference distribution 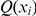. It is clear that the observed distribution eventually approaches the reference distribution over time. In this particular case the distance 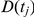 between the observed and reference distributions takes the form
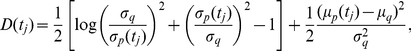
(3)where 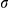 and 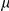 are the standard deviation and mean value of the step length, and the subscripts *p* and *q* correspond to the observed and reference distributions, respectively. The values of 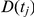 for a single tuna released after surgery are plotted in Figure 2. Though the dispersion term contributes little to the overall distance 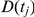 the difference in the mean values between the distributions leads to a dominant contribution to 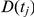 from the signal term. Since the step-length is directly correlated with the tuna's speed, one could infer that behavior of a newly tagged tuna is considerably altered due to various factors of stress.

Also evident in Figure 2 is a periodic increase in the tuna's speed, at times corresponding to the moment just before sunrise on each day. Further inspection reveals that these signal spikes are offset by about 20 minutes to the earlier side of sunrise, a result that is consistent with a previous study [21]. In the latter study, it was discovered that both the deepest portion of the dives and the most rapid changes in depth are precisely timed with respect to sunrise, with spike dives occurring at times corresponding to a sun elevation of about 30 minutes before sunrise.

**Figure 3:**
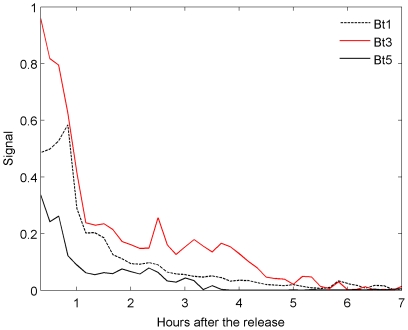
Comparison of relative entropy distributions. Signal components of the relative entropy for three bluefin tuna. In each case the behavior is significantly altered for approximately 4–6 hours.

In Figure 3 we plot the signal component of the relative entropy for three tuna fish. Large values of this term indicate that the tuna's behavior is considerably modified by distress from capture and the physiological impacts of the attachments. As the tuna recovers from the distress of capture and stress, the signal term gradually returns to zero as the observed distribution converges with equilibrium. In our case, the tuna's behavior appears to recover approximately 4–6 hours after release.

**Figure 4:**
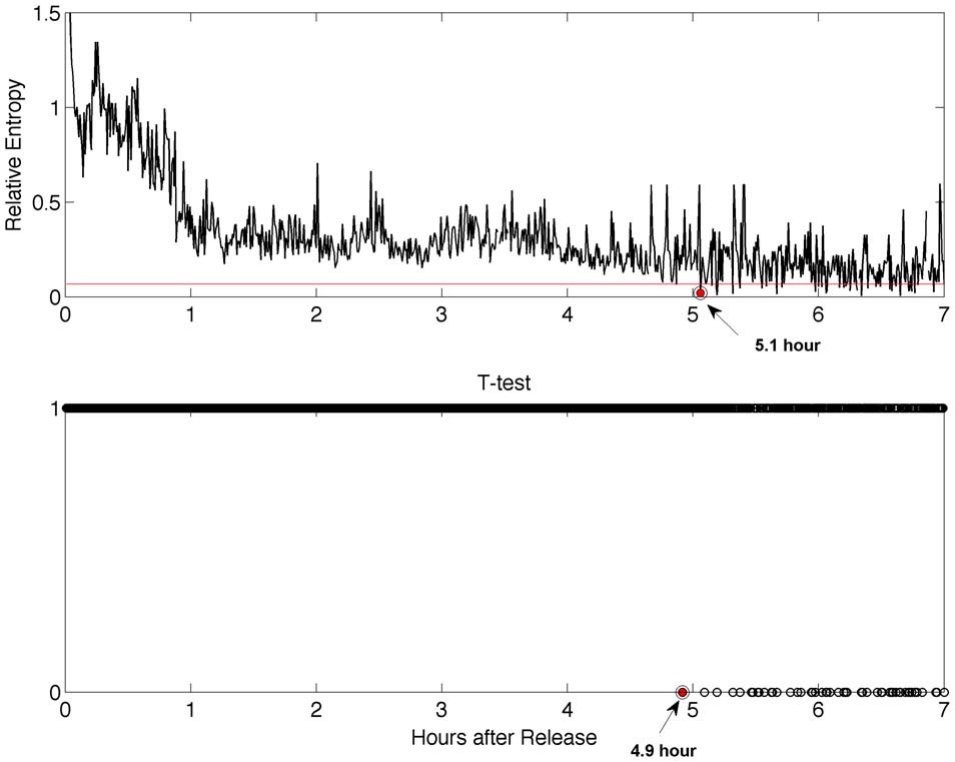
Comparison of relative entropy and *t*-test distributions. Relative entropy (top), calculated over the first seven hours after release. We determine that the distributions are indistinguishable when the relative entropy reaches a value of 0.069 (red horizontal line), first obtained after 5.1 hours (top). The *t*-test (bottom), can be used to determine that the observed and reference distributions are indistinguishable after 4.9 hours, to a 95% confidence level.

In order to demonstrate that a tuna's behavior is no longer affected by trauma, we must establish the point at which the observed distribution is the “same” as the reference distribution. Although the similarity of any two distributions is arbitrary, the previously discussed *t*-test is often used to establish when two distributions are indistinguishable, for example to a 95% confidence level. The concept of distance, as defined by the relative entropy, can also be applied as a means of distinguishing the similarity of the two distributions. In Figure 4 we plot the relative entropy in bins of 30-second intervals over the first seven hours, along with a calculation of the *t*-test for comparison. As discussed in Appendix S1, we solve the relative entropy for a *t*-value of 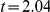, which corresponds to a confidence level of 95% for a sample with 30 degrees of freedom. We find that the distributions are indistinguishable when the relative entropy reaches a value of 0.069, first obtained after 5.1 hours as shown in Figure 4A. According to the *t*-test, the observed and reference distributions become indistinguishable after 4.9 hours, shown in Figure 4B.

## Discussion

We show how methods from information theory can be used to quantify the gain in information provided by a newly measured observation, relative to a known reference distribution. This measure, which serves as an effective distance between the observed and reference distributions, is called the relative entropy. To demonstrate the utility of these methods, we have analyzed the speed distributions of Pacific bluefin tuna over a 48-hour time span after capture and release. The reference distribution, which serves as a model of the tuna's baseline behavior, was constructed by averaging the probability density function of step-lengths over all recorded time intervals. The speed distributions, when measured immediately after a tuna's release, provide a means of observing stress-induced fluctuations in behavior. The departure of the observed behavior from baseline behavior was assessed by computing the relative entropy of the distributions, from which we discovered that the tuna's behavior is clearly modified by the process of tagging and release. In this case, the resulting modification in behavior is due primarily to a difference in the central tendencies of the two distributions, and thus the relative entropy is dominated by the signal term. We found that the modified behavior regresses to the baseline behavior after approximately 5 hours, corresponding to the first bin in which the observed distribution becomes indistinguishable from equilibrium to a 95% confidence level.

In this analysis, the relative entropy was calculated on the assumption that both the observed and reference distributions are described by Gaussian functions. For the case of Gaussian distributions, the relative entropy can be decomposed into a dispersion and signal component, the former of which depends on the variance of the distributions, the latter of which depends only on their mean value. Since the relative entropy is dominated by a difference in the mean values of each distribution, rather than a difference in their variances, it should be noted that our *t*-test was carried out explicitly for the case of two distributions with equal variances. Since the relative entropy captures information from all higher-order moments, it is no surprise that our result of 5.1 hours is slightly larger than the value of 4.9 hours determined from the *t*-test alone. In fact, a simple example serves to illustrates why the relative entropy is a more powerful test statistic in general: suppose one observes a single variable with zero variance, while the accompanying reference distribution for this variable has the exact same mean value with unit variance. In this case, the *t*-value for these two distributions is zero, and thus the signal component is also zero. Yet the behavior of the observed variable is considerably different from that of the reference distribution, namely because it possesses zero variance while the latter does not. The significance of the relative entropy is apparent: in order to accurately measure the difference between two distributions, it is necessary to include higher-order moments.

As mentioned in the Methods section, the relative entropy is invariant under a change of coordinate systems. To see why this is important, note that the step-length distributions used in this analysis are measured in units of distance. In order to make a quantitative statement about the speed of the tuna, we must in principle perform a coordinate transformation from one basis of units to another. However, since the relative entropy is invariant under such transformations, we are guaranteed that the results in the new basis are identical.

Relative entropy techniques can be used to study behavior patterns that are modified by other factors, such as water temperature, exposure to sunlight, etc. For example, in this study we discovered a periodic variation in the tuna's speed corresponding to the moment before sunrise on each day. Most likely such a fluctuation in the tuna's diving pattern is due to changes in ambient light during sunrise, a hypothesis supported by evidence from other analyses. In captive bluefin tuna, it was observed a high mortality of juveniles as a result of the fish buffeting the tank and net-pen at sunrise [22]. In the previous study it was also found that this phenomenon is caused by visual disorientation due to an incompatibility of the retina to adapt to changes in ambient light intensity. Kitagawa *et al*. [23] analyzed time-series data for depth, and reported that bluefin tuna display distinct patterns in their vertical movement at sunrise and sundown. It has also been reported that juvenile bluefin tuna make sharp descents and ascents, called spike dives, around sunrise and sunset each day [21]. Willis found that these spike dives are offset by about 30 minutes on the darker side of each sunrise or sunset, which is consistent with the results of our analysis.

There is an abundance of opportunities in which relative entropy techniques can be applied. The relative entropy is a robust and powerful method for quantifying time-dependent differences between observed data and equilibrium. Although the techniques introduced in this analysis were developed specifically for the case of Gaussian distributions, the authors soon hope to demonstrate the utility of relative entropy techniques in the context of generalized non-Gaussian distributions.

## Supporting Information

Appendix S1 **Relation Between** ***t*** **-value and Relative Entropy.**
(DOC)
