# Supplementary material for: Employing Relative Entropy Techniques for Assessing Modifications in Animal Behavior
Source: PLoS One. 2011 Dec 2;6(12):e28241. doi: 10.1371/journal.pone.0028241 (PMC3229534; doi:10.1371/journal.pone.0028241)
Supplement: Appendix S1 — Relation Between t -value and Relative Entropy. (DOC) [file pone.0028241.s001.doc]

**Supporting Information**

**Appendix S1**

Minoru Kadota^*, 1^, Eric, J. White^2^, Shinsuke Torisawa^1^, Kazuyoshi Komeyama^3^, Tsutomu Takagi^1^

^1^Department of Fisheries, Faculty of Agriculture, Kinki University, Naka-machi, Nara, Japan

^2^Department of Physics, University of Cincinnati, Clifton Ct. Cincinnati OH,U.S.A.

^3^Faculty of Fisheries, Kagoshima University, Shimoarata Kagoshima, Japan

**Relation Between *t*-value and Relative Entropy**

The *t*-test is one of the most widely used methods for determining the significance to which a single sample is representative of a known population with specified mean. Assuming that a sample follows a standard normal distribution under the null hypothesis, the statistic for testing the significance against a population with mean $m$ is given by

.

where $\bar{x}$ is the sample mean, $s$ is the standard deviation of the sample, and *n* is the size. In this study we assume the population variances of each distribution are equal, in order to satisfy the principle of pooled variance. Using the observed time-dependent mean $m_{p}$, along with the reference mean $m_{q}$, we used the *t*-test to establish the first point in time at which the observed data becomes statistically indistinguishable from the reference distribution. Assuming each distribution has equal variance ($s_{q}$=$s_{q}$), *t* statistic for our distributions becomes

. (1)

For the case of Gaussian distributions, we showed that the relative entropy can be decomposed into a dispersion and signal term:

.　　　 　　　(2)

The test statistic in (1) is related to the signal component of the relative entropy by

*Signal* $=\frac{t^{2}}{2n}$. (3)

In other words, assuming that the dispersion term is negligible, we can solve for the *t*-value in terms of the total relative entropy. In the Results section, we used this relation to solve for the threshold value of 0.069. We then used this value to find the corresponding time of 5.1 hours at which the observed distribution could be considered the same as the reference distribution ($t=2.04$,$n=30$). From a relative entropy perspective, the mean values of both the observed and reference distributions play an important role in determining the amount of information gained from an observation.
